# Supplementary material for: Epigenetic regulation of lateralized fetal spinal gene expression underlies hemispheric asymmetries
Source: eLife. 2017 Feb 1;6:e22784. doi: 10.7554/eLife.22784 (PMC5295814; doi:10.7554/eLife.22784)
Supplement: Supplementary file 1. — (A) Description of tissue samples. (B) RNA quality measurements. (C) DNA quality measurements. (D) RNA read statistics. (E) DNA read statistics. (F) Methylation report. (G) Top 25 asymmetrically expressed genes per sample. DOI: http://dx.doi.org/10.7554/eLife.22784.011 [file elife-22784-supp1.docx]

**Supplementary Files**

Supplementary File 1A: Description of tissue samples

| Week | Sample | Week + days | Application of  mifepristone | Time [mm:ss] |
| --- | --- | --- | --- | --- |
| 8 PC | I | 7 + 6 | No | 07:50 |
|  | II | 8 + 4 | No | 12:20 |
| 10 PC | III | 9 + 5 | No | 11:00 |
|  | IV | 10 + 0 | No | 09:10 |
| 12 PC | V | 11 + 2 | Yes | 13:40 |
|  | VI | 11 + 6 | Yes | 16:40 |

Supplementary File 1B: RNA quality measurements

| Week | Sample | Side | Quality measurements (RUB) | | | Quality measurements (GATC) | | | | |  |
| --- | --- | --- | --- | --- | --- | --- | --- | --- | --- | --- | --- |
|  |  |  | Concen-tration [ng/µl] | Purity [A_260_/A_280_] | Amount of RNA [µg] | | Concen-tration [ng/µl] | Amount of RNA [µg] | RIN | rRNA ratio [28S/18S] | |
| 8  PC | I | Left | 38.25 | 1.97 | 1.61 | | 32.30 | 1.36* | 5.6 | 2.1 | |
|  |  | Right | 27.82 | 2.00 | 1.17 | | 28.45 | 1.18* | 4.7 | 2.0 | |
|  | II | Left | 119.53 | 1.99 | 5.02 | | 79.33 | 3.34 | 6.8 | 1.6 | |
|  |  | Right | 72.12 | 1.98 | 3.03 | | 84.24 | 3.54 | 7.3 | 2.3 | |
| 10 PC | III | Left | 58.68 | 2.00 | 2.46 | | 53.30 | 2.24 | 3.5 | 1.6 | |
|  |  | Right | 51.37 | 2.01 | 2.16 | | 36.12 | 1.52* | 4.6 | 1.5 | |
|  | IV | Left | 249.58 | 2.04 | 10.48 | | 201.07 | 8.44 | 6.6 | 1.5 | |
|  |  | Right | 149.96 | 1.99 | 6.30 | | 165.39 | 6.94 | 6.1 | 1.7 | |
| 12 PC | V | Left | 92.51 | 2.00 | 3.89 | | 100.26 | 4.22 | 2.8 | 1.7 | |
|  |  | Right | 47.55 | 1.98 | 2.00 | | 58.74 | 2.46 | 4.3 | 1.9 | |
|  | VI** | Left | 49.78 | 1.85 | 2.09 | | 32.30 | 1.36* | 1.1 | 0 | |
|  |  | Right | 86.67 | 1.96 | 3.64 | | 28.45 | 1.18* | 1.1 | 0 | |

* The amount of RNA was not sufficient for mRNA and microRNA sequencing, so no microRNA sequencing was performed in order to enable mRNA sequencing.

** Sample VI did not pass quality control and was not further processed

Supplementary File 1C: DNA quality measurements

| Week | Sample | Side | Concentration [ng/µl] | Purity [A_260_/A_280_] | Amount of DNA [µg] |
| --- | --- | --- | --- | --- | --- |
| 8  PC | I | Left | 179.75 | 1.85 | 2.69 |
|  |  | Right | 209.08 | 1.87 | 3.14 |
|  | II | Left | 321.72 | 1.87 | 3.22 |
|  |  | Right | 272.37 | 1.86 | 2.72 |
| 10  PC | III | Left | 451.98 | 1.86 | 4.52 |
|  |  | Right | 327.73 | 1.86 | 3.28 |
|  | IV | Left | 485.38 | 1.85 | 4.85 |
|  |  | Right | 242.45 | 1.87 | 3.64 |
| 12  PC | V | Left | 496.01 | 1.88 | 4.96 |
|  |  | Right | 240.91 | 1.89 | 2.41 |

Supplementary File 1D: RNA read statistics

| Week | Sample | Side | Total Reads | QC passed reads | % Passed | Mapped reads | % Mapped |
| --- | --- | --- | --- | --- | --- | --- | --- |
| 8  PC | I | Left | 138,364,454 | 133,215,718 | 96.3 | 128,422,198 | 96.4 |
|  |  | Right | 156,787,326 | 148,278,816 | 94.6 | 143,107,356 | 96.5 |
|  | II | Left | 153,431,660 | 145,032,810 | 94.5 | 141,227,019 | 97.4 |
|  |  | Right | 167,209,892 | 161,440,222 | 96.5 | 152,747,761 | 94.6 |
| 10 PC | III | Left | 124,939,458 | 120,500,696 | 96.4 | 115,757,503 | 96.1 |
|  |  | Right | 170,552,644 | 163,290,090 | 95.7 | 158,166,665 | 96.9 |
|  | IV | Left | 154,704,654 | 148,006,032 | 95.7 | 143,653,484 | 97.1 |
|  |  | Right | 130,817,636 | 125,353,002 | 95.8 | 121,841,246 | 97.2 |
| 12 PC | V | Left | 95,466,628 | 92,592,902 | 97.0 | 24,356,396 | 26.3 |
|  |  | Right | 139,152,284 | 134,489,354 | 96.6 | 35,694,631 | 26.5 |

Supplementary File 1E: DNA read statistics

| Week | Sample | Side | Total Reads | QC passed reads | % Passed | Mapped reads | % Mapped |
| --- | --- | --- | --- | --- | --- | --- | --- |
| 8  PC | I | Left | 157,131,898 | 135,957,542 | 86.5 | 98,904,834 | 72.7 |
|  |  | Right | 116,438,904 | 98,145,354 | 84.3 | 71,949,168 | 73.3 |
|  | II | Left | 144,313,254 | 134,707,790 | 93.3 | 100,057,784 | 74.3 |
|  |  | Right | 149,788,924 | 140,866,420 | 94.0 | 107,706,526 | 76.5 |
| 10 PC | III | Left | 190,838,816 | 178,029,952 | 93.3 | 130,557,346 | 73.3 |
|  |  | Right | 169,157,704 | 157,992,478 | 93.4 | 119,759,046 | 75.8 |
|  | IV | Left | 133,073,726 | 122,808,294 | 92.3 | 94,880,640 | 77.3 |
|  |  | Right | 151,718,790 | 140,366,174 | 92.5 | 42,861,510 | 30.5 |
| 12 PC | V | Left | 219,803,208 | 206,451,844 | 93.9 | 156,865,926 | 76.0 |
|  |  | Right | 172,963,332 | 162,929,394 | 94.2 | 122,763,788 | 75.3 |

Supplementary File 1F: Methylation report

| Week | Sample | Side | Unique CpGs (raw) | Unique CpGs (filtered) | Average Methylation Level | Bisulphite conversion rate | Average CpG coverage |
| --- | --- | --- | --- | --- | --- | --- | --- |
| 8  PC | I | Left | 1,901,233 | 1,771,838 | 68.15% | 99.1% | 105.2 |
|  |  | Right | 1,757,660 | 1,602,695 | 63.85% | 99.3% | 84.0 |
|  | II | Left | 1,512,131 | 1,359,787 | 59.33% | 99.3% | 119.8 |
|  |  | Right | 1,805,290 | 1,672,820 | 64.46% | 99.3% | 128.8 |
| 10 PC | III | Left | 1,529,610 | 1,374,690 | 59.36% | 98.9% | 173.4 |
|  |  | Right | 1,302,963 | 1,129,590 | 54.66% | 99.1% | 172.6 |
|  | IV | Left | 1,195,800 | 1,037,570 | 53.91% | 99.1% | 152.6 |
|  |  | Right | 1,520,897 | 1,323,650 | 57.97% | 99.4% | 51.7 |
| 12 PC | V | Left | 1,705,992 | 1,564,450 | 63.93% | 99.3% | 158.0 |
|  |  | Right | 1,879,865 | 1,760,330 | 66.52% | 99.4% | 145.7 |

**Supplementary File 1G:** Top 25 asymmetrically expressed genes per sample

|  | 8 weeks PC | | | |
| --- | --- | --- | --- | --- |
|  | I | | II | |
|  | Left | Right | Left | Right |
| 1 | STK35 | RPH3A | CDR1 | DHRS2 |
| 2 | CBFA2T2 | SLC39A11 | SEMA6D | MT2A |
| 3 | GPR125 | LRRIQ3 | GRIA2 | TNFRSF12A |
| 4 | MCU | LTA4H | CDR1 | MYBL2 |
| 5 | TASP1 | PLEKHH2 | MYO16 | TK1 |
| 6 | RAB28 | NHSL1 | LARP4 | TGFB1 |
| 7 | FAM65C | EFHB | NEFM | PTRF |
| 8 | COL3A1 | VRK2 | NRXN1 | TYMS |
| 9 | TENC1 | HS3ST1 | ANKRD36BP2 | SLC1A5 |
| 10 | SHROOM4 | ALS2CR12 | CCSER1 | KRT18 |
| 11 | ZRANB2-AS2 | NOMO2 | RALYL | FOXM1 |
| 12 | GPI | IL1RAPL1 | SYT4 | LINC00152 |
| 13 | NIPAL2 | SDCBP2 | PTPRD | C1QL1 |
| 14 | PLEKHH1 | CA8 | ATG16L1 | HSPB1 |
| 15 | XYLB | ADAMTS13 | VSNL1 | LGALS1 |
| 16 | ARHGAP31 | TACR3 | VSTM2A | BCAM |
| 17 | COL1A2 | STARD3 | GRIK2 | ACTN1 |
| 18 | CACNA1D | C5orf64 | LRRN3 | PDLIM7 |
| 19 | MLIP | ZBTB49 | SLC17A6 | MAFK |
| 20 | IGSF9 | TMLHE-AS1 | HIST1H3C | MAP2K3 |
| 21 | LY6G5B | NEDD9 | TMOD2 | RECQL4 |
| 22 | CIT | ANXA4 | FSTL5 | MYO1C |
| 23 | ADAMDEC1 | PODXL | INA | AXL |
| 24 | PLBD2 | ADAMTS5 | PDE4B | VGF |
| 25 | MCM9 | PRR4 | PHYHIPL | TAGLN2 |

|  | 10 weeks PC | | | | 12 weeks PC | |
| --- | --- | --- | --- | --- | --- | --- |
|  | III | | IV | | V | |
|  | Left | Right | Left | Right | Left | Right |
| 1 | NFIA | MZT2A | GPR125 | CRH | - | SLC6A13 |
| 2 | PYY | TENC1 | LBX1 | FOXP2 | - | MYO1D |
| 3 | EP400NL | KIAA1462 | CHRNE | ETV1 | - | GJB1 |
| 4 | PLAGL2 | PLEKHH2 | C5orf58 | SULF1 | - | ZNF257 |
| 5 | ZNF396 | PPP1R3F | LHX9 | GLI3 | - | - |
| 6 | RBM26-AS1 | NIPSNAP3B | PROSER2 | EYA4 | - | - |
| 7 | SLC38A4 | GJC3 | BOC | CPA6 | - | - |
| 8 | POLR2M | COL27A1 | ARHGAP15 | LPL | - | - |
| 9 | MIR4500HG | TLX3 | DRGX | SRPK2 | - | - |
| 10 | DTHD1 | FAM172A | PAX3 | PIP5K1B | - | - |
| 11 | ARHGAP22 | DEPDC1 | MAFA | CD36 | - | - |
| 12 | TMEM72-AS1 | BNC2 | MAGEC3 | HMX3 | - | - |
| 13 | GGACT | ANO1 | LMX1B | KCNG1 | - | - |
| 14 | CHRM5 | GBX1 | POM121L4P | UTS2 | - | - |
| 15 | RPGR | PRHOXNB | NPY | EYA1 | - | - |
| 16 | IGSF9B | LMX1B | ZIC3 | NBPF14 | - | - |
| 17 | SULF1 | DRGX | EBF3 | FMO1 | - | - |
| 18 | ZNF710 | CLUHP3 | EBF1 | CORIN | - | - |
| 19 | XKR9 | - | NPPC | IGF1 | - | - |
| 20 | TAF3 | - | ANK1 | CRHBP | - | - |
| 21 | C21orf49 | - | GPC3 | NKX6-1 | - | - |
| 22 | SLC27A6 | - | SAMD12 | IL13RA2 | - | - |
| 23 | TATDN2 | - | TKTL1 | BMP3 | - | - |
| 24 | ADAM5 | - | UNCX | PI16 | - | - |
| 25 | EOGT | - | TACR1 | POU4F1 | - | - |
